# Validation of a size exclusion method for concomitant purification and formulation of peptide radiopharmaceuticals

**DOI:** 10.1186/s41181-024-00254-2

**Published:** 2024-03-21

**Authors:** Sebastian Martin, Lennard Wendlinger, Alexandra Litvinenko, Radmila Faizova, Margret Schottelius

**Affiliations:** 1 Translational Radiopharmaceutical Sciences, Department of Nuclear Medicine and Department of Oncology, Centre Hospitalier Universitaire Vaudois (CHUV) and University of Lausanne (UNIL), Rue du Bugnon 25A, Agora, Lausanne, CH-1011 Switzerland; 2 AGORA, Pôle de recherche sur le cancer, Lausanne, 1011 Switzerland; 3 SCCL Swiss Cancer Center Leman, Lausanne, 1011 Switzerland

**Keywords:** Size-exclusion cartridge, Peptide radiopharmaceutical, Tracer, Purification, Sephadex G10

## Abstract

**Background:**

Both in clinical routine and in preclinical research, the established standard procedure for the final purification of radiometal-labeled peptide radiopharmaceuticals is cartridge-based reversed-phase (RP) solid phase extraction (SPE). It allows the rapid and quantitative separation of the radiolabeled peptide from hydrophilic impurities and easy integration into automated synthesis procedures. However, product elution from RP cartridges necessitates the use of organic solvents and product recovery is sometimes limited. Thus, an alternative purification method based on commercially available size exclusion cartridges was investigated.

**Results:**

Since most peptide radiopharmaceuticals have a molecular weight > 1 kDa, Sephadex G10 cartridges with a molecular size cut-off of 700 Da were used for the final purification of a broad palette of ^68^Ga-, ^64^Cu- and ^99m^Tc-labeled experimental peptide radiotracers as well as the clinically relevant ligand PSMA-617. Results (radiochemical purity (RCP, determined by ITLC), recovery from the solid support) were compared to the respective standard RP-SPE method. Generally, retention of unreacted ^68^Ga, ^64^Cu and ^99m^Tc salts on the G10 cartridges was quantitative up to the specified elution volume (1.2 mL) for ^68^Ga and ^99m^Tc and 99.6% for ^64^Cu. Even at increased elution volumes of 1.5-2 mL, RCPs of the eluted ^68^Ga- and ^99m^Tc -radiopeptides were > 99%. For all peptides with a molecular weight ≥ 2 kDa, product recovery from the G10 cartridges was consistently > 85% upon respective adjustment of the elution volume. Product recovery was lowest for [^68^Ga]Ga-PSMA-617 (67%, 1.2 mL to 84%, 2 mL). The pH of the final product solution was found to be volume-dependent (1.2 mL: pH 6.3; 1.5 mL: pH 5.9; 2 mL: pH 5.5). Notably, the G10 cartridges were reused up to 20 times without compromising performance, and implementation of the method in an automated radiosynthesis procedure was successful.

**Conclusions:**

Overall, size exclusion purification yielded all peptide radiopharmaceuticals in excellent radiochemical purities (> 99%) in saline within 10–12 min. Although product recovery is marginally inferior to classical SPE purifications, this method has the advantage of completely avoiding organic solvents and representing a cost-effective, easy-to-implement purification approach for automated radiotracer synthesis.

**Supplementary Information:**

The online version contains supplementary material available at 10.1186/s41181-024-00254-2.

## Background

The widely established standard labeling protocols for ^68^Ga-, ^64^Cu- or ^99m^Tc-labeling of chelator functionalized peptides usually provide excellent radiolabeling yields, with frequently > 97% of incorporated radionuclide. Nevertheless, both in clinical routine production and in preclinical research, the preparation of radiometal-labeled peptide radiopharmaceuticals almost always involves a last purification step using (reversed phase (RP)) solid phase extraction (SPE) cartridges (Neels et al., 2018; Decristoforo et al., 2007; Hennrich & Benešová, 2020; Hennrich & Eder, 2022; Hennrich & Eder, 2021). This step ensures quantitative removal of free radionuclide or radionuclide colloids and of non-physiological, potentially toxic labeling buffer components, such as HEPES (Nelson et al., 2022). However, product elution requires organic solvents like ethanol (Decristoforo et al., 2007; Hennrich & Eder, 2021; Nelson et al., 2022). Additionally, acidic conditions may prove indispensable to obtain sufficient product recovery from the solid phase (Robu et al., 2017). This, in turn, entails additional processing steps, such as solvent evaporation, neutralization or dilution to meet the specifications for injectable solutions (pH 4–8, ethanol ≤ 10% v/v) (Hennrich & Eder, 2021; Serdons et al., 2008). Although small amounts of ethanol are accepted in the final formulation of peptide radiopharmaceuticals, the presence of ethanol can lead to pain and haemolysis at the injection site (Serdons et al., 2008). On the other hand, evaporation of the organic solvent by heat under nitrogen flow is time consuming (10–20 min) and leads to a loss of activity, especially when working with short-lived radioisotopes such as ^68^Ga. In some cases, another drawback of SPE purification consists in the poor recovery of the final product from the solid support. This has been shown for [^68^Ga]Ga-DOTA-TBIA101 (Mokaleng et al., 2015), for which low recovery limited the overall yield of the final product.

To circumvent the complications associated with the use of RP-SPE cartridges, we investigated the use of size exclusion chromatography (SEC) cartridges as an alternative, organic-solvent-free purification method for radiometal-labeled peptide radiopharmaceuticals. Since most peptide radiotracers have a molecular weight > 1 kDa, Sephadex G10 cartridges with a molecular weight cut-off of 700 Da (for a sample volume of 1 mL) were chosen for this study. After assessing the retention capacities of the material for free ^68^GaCl_3_, ^64^CuCl_2_ and ^99m^TcO_4_^−^, the size exclusion purification method was investigated for the post-labeling workup of a variety of ^68^Ga-, ^64^Cu- and ^99m^Tc-labeled peptide radiopharmaceuticals in a molecular weight range of 1–3 kDa. [^68^Ga]Ga-PSMA-617 was included as a clinically relevant reference. Key parameters such as radiochemical purity (RCP) after purification and recovery from the cartridge material were quantified and compared to the respective results obtained by SPE purification. In a last step, the SEC-based purification was implemented in a semi-automated synthesis protocol.

## Results

**Fig. 1:**
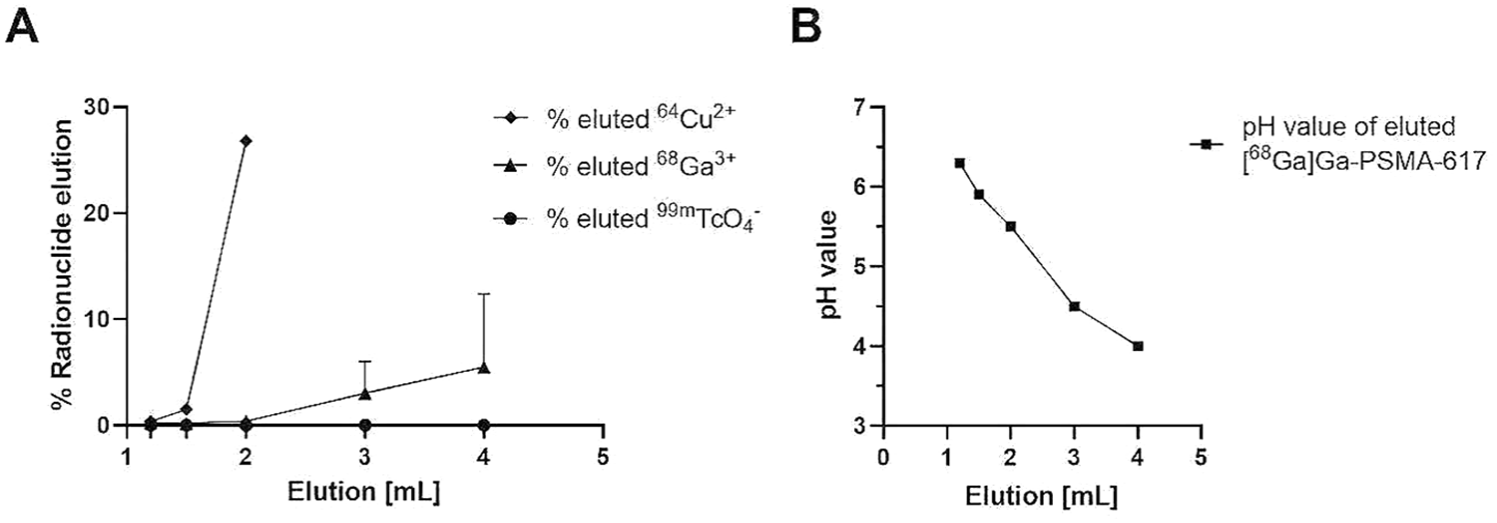
(**A**) Retention capacity of the G10 size exclusion cartridge for the free ^68^Ga, ^64^Cu and ^99m^Tc-radionuclide salts as present in the radiolabeling mixture. Saline (0.9% NaCl) was used for elution. (*B*) Exemplary correlation between elution volume and pH of the final product [^68^Ga]Ga-PSMA-617 in labeling buffer (1 M NaOAc, pH 4.5)

The results obtained for the retention capacity of the G10 cartridges for the free ^68^Ga-, ^64^Cu- and ^99m^Tc-radionuclide salts are summarized in Fig. 1A. All reaction mixtures were applied to the cartridge in a fixed volume of 0.5 mL and in the respective radiolabeling buffer (no colloid formation). In the case of ^68^Ga, quantitative retention of the free radionuclide was observed for a 1.2 mL elution volume, and only 0.4 ± 0.2% of free ^68^Ga was eluted, when the elution volume was increased to 2 mL. In contrast, for ^64^Cu, 0.4% of the activity were already eluted with 1.2 mL 0.9% NaCl, increasing to > 1% and > 25% of the initial activity, respectively, when elution volumes of 1.5 and 2 mL were used. Interestingly, ^99m^Tc-pertechnetate showed quantitative retention on the size exclusion cartridge, both when applied in radiolabeling buffer and as pure generator eluate (in saline), even when the elution volume was increased to 4 mL. Retention of freshly generated ^99m^Tc-tin colloid was slightly less efficient (see materials and methods). While 98% of the^99m^Tc-tin colloid remained trapped on the G10 column, 1.0 ± 0.7% were eluted using a 2 mL elution volume. For ^68^GaOH_3_ colloid, app. 50% of the activity were eluted using an elution volume of 1.2 mL.

Next, the buffer exchange capacity of the used G10 cartridges was investigated by pH measurements of the eluate at different elution volumes. The pH values were determined both by pH paper strips and a pH electrode. After the radiolabeling of PSMA-617 in sodium acetate buffer (1 M, pH = 4.5) as an exemplary tracer, the pH of the eluate remained stable at pH 6.2 ± 0.1, when 1.2 mL of 0.9% NaCl (pH 6.3) were used for product elution. A slight decrease in pH was observed at an elution volume of 1.5 mL (pH 5.9 ± 0.1), while increasing the elution volume to 2 mL decreased the pH to 5.5 (see Fig. 1B). This decrease is due to the co-elution of the acidic labeling buffer. Thus, to achieve a physiological pH of the final product formulation, 300 µL of phosphate buffer (0.5 M, pH 8) had to be added to the eluate. In contrast, since ^99m^Tc-labeling of PSMA-HSG was carried out in phosphate buffer (pH 7.5), the pH of the column eluate remained neutral throughout the elution (1.2-2 mL 0.9% NaCl) of the radiolabeled peptide.

After this initial validation of the G10 cartridge-based SEC method for the size exclusion purification of radiometal-labeled peptides, its suitability for the post-labeling work-up of several ^68^Ga-, ^64^Cu- and ^99m^Tc-labeled peptides in a molecular weight range from 1 to 3 kDa was investigated. The used peptide radiopharmaceuticals include the reference compound [^68^Ga]Ga-PSMA-617 (app. 1 kDa), the CCR5-targeted ligands including unpublished compounds [^68^Ga]Ga-DOTA-Rap-103 (app. 1 kDa) and its trimeric counterparts [^68^Ga]Ga-TRAP-103 (app. 3 kDa), [^64^Cu]Cu-TRAP-103 (app. 3 kDa) and [^64^Cu]Cu-NO-Y-103 (app. 2.5 kDa), the PD-1 targeted peptides [^68^Ga]Ga-mPep-DOTA (app. 2 kDa) and [^68^Ga]Ga-hPep-DOTA (app. 2 kDa) (Hu et al., 2020) as well as the novel hybrid PSMA-tracer [^99m^Tc]Tc-PSMA-HSG (app. 2 kDa) (manuscript in preparation ). The structures of all ligands are provided in the supplementary information (Supp. Figure S1-S7).

The most important parameters in this part of the evaluation were the recovery of the radiolabeled peptide from the size exclusion material as well as the radiochemical purity (RCP) of the final product. For an elution volume of 1.2 mL, RCPs > 99% (as determined by ITLC or HPLC) were observed for all peptides, independently of the radionuclide or the molecular weight of the peptide precursor. An increase of the elution volume to 2 mL still provided the respective peptide radiopharmaceuticals in > 99% RCP (see Supp. Fig. S8-S11), allowing the adjustment of the elution volume to the retention behaviour of the specific peptide.

**Fig. 2:**
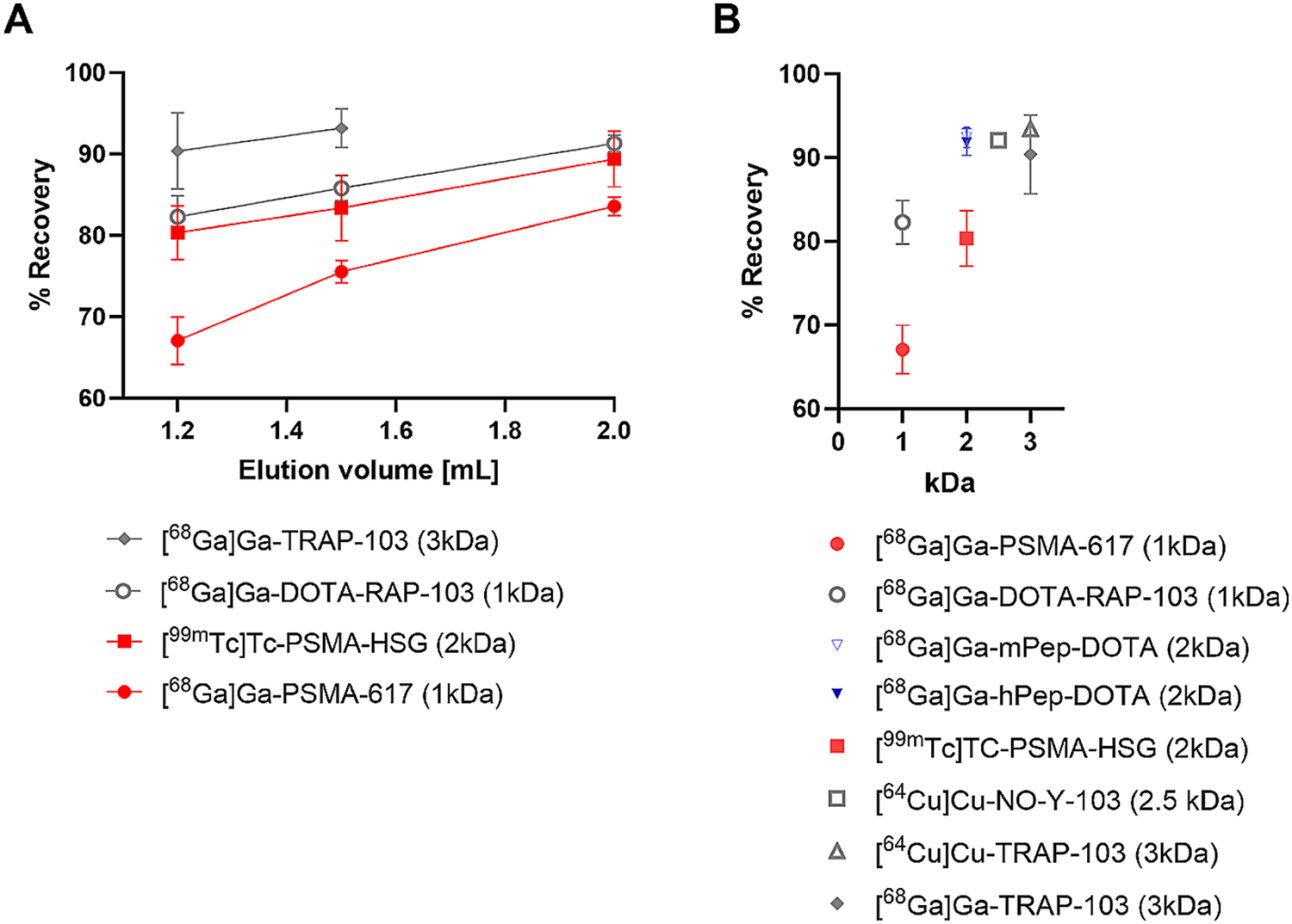
Assessment of the recovery using a G10 cartridge. Gray: radiotracers with neutral binding motif; Red: radiotracers with anionic binding motif; Blue: radiotracers with cationic binding motif. (**A**) Recovery of selected peptide radiopharmaceuticals as a function of elution volume. (**B**) Recovery of the investigated peptide radiopharmaceuticals at a fixed elution volume of 1.2 mL as a function of molecular weight

That this is highly relevant in terms of recovery from the SEC cartridge, is shown in Fig. 2A. For all peptides investigated, increasing the elution volume led to improved tracer recoveries from the SEC cartridge. This effect was much more pronounced for the low-molecular-weight compounds [^68^Ga]Ga-DOTA-RAP-103 (1 kDa) and [^68^Ga]Ga-PSMA-617 (1 kDa) compared to their higher-molecular-weight counterparts [^68^Ga]Ga-TRAP-103 (3 kDa) and [^99m^Tc]Tc-PSMA-HSG (2 kDa), respectively. Recoveries of the trimeric RAP-103-analogs [^68^Ga]Ga-TRAP-103, [^64^Cu]Cu-TRAP-103 and [^64^Cu]Cu-NO-Y-103 were all comparable and high at 1.2 mL elution volume (91.1, 93.5 and 92.1%, respectively; see Fig. 2B), while an elution volume of 2 mL was required for a similar recovery of the structurally related monomer [^68^Ga]Ga-DOTA-RAP-103 (Fig. 2A).

To further improve recoveries, alternative elution buffers (saline containing different additives, see Supp. Table S1) were used for the SEC purification of the exemplary compounds [^68^Ga]Ga-PSMA-617 and [^68^Ga]Ga-TRAP-103. However, neither the addition of 5% ethanol nor the addition of the chaotropic agent guanidine (50 mM) nor of 0.5% of the non-ionic surfactant polysorbate 80 had any noteworthy influence of tracer recovery.

Finally, the recoveries obtained using the G10 cartridge based purification method were compared to the most-commonly used RP-SPE purification method for selected compounds ([^68^Ga]Ga-PSMA-617, [^68^Ga]Ga-TRAP-103, [^68^Ga]Ga-mPep-DOTA and [^99m^Tc]TC-PSMA-HSG). For the elution of [^68^Ga]Ga-PSMA-617 and [^68^Ga]Ga-TRAP-103, 1.5 mL of a 4:1 ethanol/water mixture was used, and for the elution of [^68^Ga]Ga-mPep-DOTA and [^99m^Tc]Tc-PSMA-HSG, 0.5 mL 0.1% TFA in acetonitrile were used. For all compounds except [^68^Ga]Ga-PSMA-617, product recovery from the C18 cartridge material under the chosen conditions was 3–10% higher than from the G10 size exclusion cartridge. For [^68^Ga]Ga-PSMA-617, SPE work-up afforded a significantly higher recovery of > 95%, while SEC elution using 1.5 mL 0.9% NaCl had provided a recovery of only 75.5 ± 1.4%.

To demonstrate the ease of implementation of the G10-cartridge based SEC purification method into automated radiosynthesis protocols, [^68^Ga]Ga-TRAP-103 was also synthesized and purified using a automated radiosynthesis module (Scintomics GRP series, see Supp. Figure S12) and NaCl elution of SCX-bound ^68^Ga (Mueller et al., 2012). The final automated SEC purification of the reaction mixture (1 mL) provided a product recovery of 80.0 ± 2.5% (*n* = 3) in 1.2 mL elution volume, which was comparable to the observed recovery obtained during manual synthesis using 1.0 mL reaction volume (81.9%).

## Discussion

Using commercially available SEC cartridges with a molecular cut-off of 700 kDa, we systematically investigated the purification of peptide radiotracers of different charge and size labeled with ^64^Cu, ^68^Ga, and ^99m^Tc-pertechnetate. With respect to the retention capacity of the SEC cartridge for unreacted radionuclide, only ^64^Cu showed comparably low retention by the cartridge. This could be attributed to the increased molecular size of the copper acetate complex formed in the acetate labeling buffer as a stabilized intermediate during ^64^Cu-labeling (Kyuzou et al., 2010). In contrast, the ^99m^Tc-pertechnetate in radiolabeling buffer was retained quantitatively by the size-exclusion material. This hints towards non-size effects in the retention of the ^99m^Tc-pertechnetate anion that have already been described for Sephadex material, where increasing ionic strength of eluent was found to substantially increase the retention of ^99m^Tc-pertechnetate on the size exclusion material (Harms et al., 1996). Surprisingly, the free ^68^Ga^3+^ was also retained with > 99% even when using 2 mL of elution volume, while colloidal ^68^GaOH_3_ was poorly retained on the cartridge. This is in accordance with a study using PD-10 size exclusion cartridges to separate ^68^GaOH_3_ colloids from the radiotracer ^68^Ga-DOTA-exendin-3, where (co)elution of ^68^GaOH_3_ colloids was equally observed (Brom et al., 2016). In contrast, and probably due to charge effects, colloidal ^99m^Tc-tin showed very high (> 98%), but not quantitative retention on the G10 cartridge. It is important to note, however, that usually, when widely established, optimized radiolabeling conditions are used, the formation of colloidal species is efficiently suppressed and should thus not interfere with size-exclusion-based purification methods. Overall, these combined observations allowed the adaptation of the elution volume for ^68^Ga- and ^99m^Tc-labeled peptides to higher elution volumes (2mL), which in turn resulted in improved recovery rates of the final product to > 90%, with RCPs always ≥ 99%.

For all peptides investigated, an increased elution volume consistently led to improved tracer recoveries from the SEC cartridge. According to the principle of size exclusion chromatography, larger-molecular-weight compounds elute in lower volume and sharper peaks than smaller compounds. This explains why recoveries of the trimeric RAP-103-analogs [^68^Ga]Ga-TRAP-103, [^64^Cu]Cu-TRAP-103, and [^64^Cu]Cu-NO-Y-103 were all comparable and high, while an elution volume of 2 mL was required for a similar recovery of the structurally related monomer [^68^Ga]Ga-DOTA-RAP-103. However, non-size-related structural effects also play a role in the recovery of radiometal-labeled peptide radiopharmaceuticals from the G10 cartridges used in this study. Both anionic PSMA-ligands show substantially lower recovery from the Sephadex material than their respective comparable molecular weight counterparts with neutral as well as cationic net charge. Most probably this is a result of the high number of anionic charges in the targeting moiety of the PSMA-ligands, leading to increased adsorption to the gel material due to increased hydrogen bonding as opposed to end-capped silica material used in SPE.

In summary, the SEC based purification method has distinct advantages and disadvantages over conventional SPE methods. The reusability of size exclusion cartridges for up to 20 cycles without compromising RCP and recovery of the eluted products, provides a cost-effective and sustainable alternative to single-use SPE cartridges. With respect to overall synthesis time, the presented gravity-based purification method (10–12 min) is more time-consuming than SPE purification (4–5 min). However, SEC purification represents an advantage over RP-SPE purification, especially in the preclinical tracer synthesis setting, where lengthy organic solvent evaporation and reconstitution steps can be omitted (Hörmann et al., 2022). Despite these strengths, it is crucial to acknowledge the associated minor limitations in product recovery, which have been found to be slightly superior for RP-SPE methods, as well as the dependence of product recovery on the molecular weight, charge and charge distribution of the respective peptide radiopharmaceutical. However, the major advantage of the SEC purification method presented in this study is the direct elution of the radiolabeled product in a physiological buffer or saline. Of note, direct product elution without organic solvents is also feasible using an SPE purification method based on cation exchange cartridges. For example, [^68^Ga]Ga-FAPI-046 was obtained in excellent yields and purities using cation exchange cartridge purification, with efficient retention of free ^68^Ga (Spreckelmeyer et al., 2020). However, the efficiency and performance of cation exchange-based separation methods are largely dependent on the net charge and structure of the specific tracer molecule, and the G10-based size exclusion method presented in this study benefits from a much greater versatility and broader applicability.

With respect to clinical application, the G10 cartridges require more extensive validation. Similar Sephadex-based cartridges (PD-10) have been successfully utilized to purify the radiolabeled monoclonal antibody ^211^At-BC8-B10, meeting the cGMP requirements (pyrogen-free and > 95% purity) for subsequent human trials (Spreckelmeyer et al., 2020). In principle, the present G10-based method may thus also be suited for clinical application, and since the content of organic solvent contaminants in the final radiopharmaceutical formulation is a decisive release criterion (Chi et al., 2014; Agency EM., 2019), the possibility to perform an organic-solvent-free purification represents a major simplification.

## Conclusions

The use of size exclusion cartridges for the purification of radiometal-labeled peptide radiopharmaceuticals represents a valuable alternative to conventional RP-SPE purification methods. Especially for the investigated radiotracers at a molecular weight ≥ 2 kDa, SEC purification was found to reliably provide structurally diverse peptide tracers in consistently high yields (≥ 80% recovery from the SEC cartridge) and excellent RCPs (≥ 99%) in a ready-to-inject physiological formulation. Its easy implementation into an automated synthesis protocol, its sustainability, and particularly the fact that the SEC-cartridge based tracer purification is inherently an organic-solvent-free procedure make it highly attractive for routine implementation in preclinical research and clinical tracer production.

## Methods

### Quality control

#### Thin layer chromatography

The RCP was determined by instant thin layer chromatography (ITLC) using a Scan-RAM radio-TLC scanner (LabLogic) and Laura software (LabLogic, Version 6.0.3). ITLC analyses were performed on dried ITLC-SG Glass microfiber chromatography paper coated with silica gel (Agilent Technologies, Folsom, CA 95,630). The radiotracers were analyzed by a retention factor (R_f_), reflecting the migration distance of the compound relative to the spotting line.

#### Reversed phase high performance liquid chromatography (RP-HPLC)

Upon SEC purification, selected peptide tracers were also analyzed via RP-HPLC using a Shimadzu LC-20AT Gradient HPLC system equipped with an SPD-M20A UV/VIS detector and a Gabi Na(I) well-type scintillation detector (Elysia-Raytest). Either a chromolith RP-18e, 100 × 4.6 mm (Merck KGaA, Darmstadt) column (flow rate: 2 mL/min) or a MultiKrom 100-5 C18, 150 × 4.6 mm (CS-Chromatographie Service GmbH, Langerwehe) column (flow rate: 1 mL/min) was used. Peptides were eluted using different gradients of solvent B (acetonitrile, 0.1% TFA) in solvent A (0.1% TFA in water). Specific gradients are cited in the text.

### Radiolabeling and radiotracer purification

#### ^68^Ga-radiolabeling

For ^68^Ga-labeling, a slightly modified protocol based on the method developed by Mueller et al. (2012) was used. Briefly, 10 nmol (TRAP-103) or 25 nmol (NO-Y-103, hPep-DOTA, mPep-DOTA, DOTA-RAP-103, PSMA-617) of peptide precursor (1–5 mM stock concentration) were added to 350–450 µL of NaOAc buffer (1 M, pH 4.5). The ^68^GaCl_3_ eluate from a ^68^Ge/^68^Ga generator (Eckert & Ziegler AG, activity 500 MBq) was either collected by fractionated elution, or the entire eluate (6 mL) was passed through a SCX cartridge (Waters), which had been preconditioned with 1 M HCl (1 mL) and deionized water (10 mL) and dried with air (10 mL). For the manual synthesis, the SCX cartridge was eluted with 500 µL 5 M NaCl/134 mM HCl. A fraction of the purified ^68^GaCl_3_ solution (50–100 MBq, 50–150 µL) was then used for radiolabeling, with a final volume of 0.5 mL of the total labeling mixture. The mixture was then heated to 85–90 °C for 12 min. ITLC before and after the purification was performed with a solution of NH_4_OAc buffer (1 M, pH 4.5) in methanol (1:1 v/v). (Colloidal ^68^GaOH_3_ and ^68^GaCl_3_, R_f_ = 0, ^68^Ga-labeled peptide R_f_ = 1).

#### Automated ^68^Ga-radiolabeling and purification

For the automated radiosynthesis Scintomics GRP V3 module (see Supp. Fig. S9) was used. The ^68^Ga was eluted from a SCX cartridge using 700 µL NaCl/HCl (5 M NaCl/134 mM HCl). The automated SEC purification was carried out as described, using a fixed volume of 1.2 mL of 0.9% NaCl for product elution. ITLC analysis before and after the purification was performed using the buffer system cited above.

#### Colloidal ^68^GaOH_3_

^68^GaOH_3_ was prepared similarly to Brom et al. with minor changes (2016). Briefly, 1 mL of a 1:2 (v/v) mixture of ^68^Ga generator-eluate (50–80 MBq in 0.1 N HCl) and phosphate buffer (0.2 M, pH 7.4) was heated to 90 °C for 10 min. The resulting colloidal ^68^GaOH_3_ (500 µL) was added to a G10 column and the elution was performed as described. Colloidal ^68^GaOH_3_ was verified by ITLC using 0.1 M EDTA in NH_4_OAc (0.25 M, pH 5.5) as mobile phase (R_f_^68^GaOH_3_ colloid = 0, R_f_^68^Ga-EDTA = 1). ^*64*^*Cu-radiolabeling*^64^CuCl_2_ was provided by the Arronax (Nantes, France) cyclotron facility. ^64^Cu-labeling was carried out in 450 µL of labeling buffer (0.1 M NaOAc, pH 5.5) containing 10 nmol of peptide precursor. The total reaction volume was 0.5 mL, and the radiolabeling was performed at 85 °C for 12 min. Pure ^64^CuCl_2_ solutions (for analysis of the elution profile of the radionuclide from G-10 cartridges) were prepared by adding 50 µl of eluate to the labeling buffer to achieve a final volume of 500 µL. ITLC before and after the purification was performed using 0.1 M citrate buffer (pH 5) as mobile phase. A volume of 5 µL EDTA (1 mM) was added to the sample before the analysis. (^64^CuCl_2_, R_f_ = 1, ^64^Cu-labled peptide R_f_ = 0).

#### ^99m^Tc-radiolabeling

Lyophilized kits containing 10 nmol of PSMA-HSG peptide precursor (manuscript in preparation) were prepared according to Robu et al. (2017). A volume of 0.5 mL of ^99m^Tc-pertechnetate was added to the vial and heated to 90 °C for 15 min.

Pure ^99m^Tc-pertechnetate solutions (for analysis of the elution profile of the radionuclide from G-10 cartridges) were prepared by addition of the gerenator eluate to precursor-free labelling kits. The finial pH of all preparations was 7-7.5. ITLC before and after G10 purification was performed using 2-Butanone (colloidal ^99m^TcO_2_ and ^99m^Tc-labeled peptide R_f_ = 0, ^99m^Tc-pertechnetate R_f_ = 1) and NH_4_OAc/DMF (1:1) (colloidal ^99m^TcO_2_, Rf = 0, ^99m^Tc-pertechnetate and ^99m^Tc-labeled peptide R_f_ = 1) as mobile phases to discriminate between colloidal, free ^99m^Tc-pertechnetate, and the labeled peptide.

#### Colloidal ^99m^Tc-tin

Colloidal ^99m^Tc-tin was prepared similarly to a reported Kit-preparation with minor changes (Gil Valenzuela et al., 2008). Briefly, 100 µl SnCl_2_ (0.3 mg in 0.02 N HCl) and 100 µl of a solution containing NaCl (3.6 mg) and NaF (1.0 mg) were added to an Eppendorf tube. Subsequently, ^99m^Tc-pertechnetate (2 mL, approximately 100 MBq) was added and incubated for 20 min at room temperature. The pH or the mixture was verified with pH paper strips (pH 5–6). A volume of 500 µl of this mixture was added to the G10 cartridge and the elution was performed as described. Colloidal ^99m^Tc-tin was identified by ITLC using saline as mobile phase (R_f_^99m^Tin-colloid = 0, R_f_^99m^Tc-pertechnetate = 1).

#### G10 elution protocol

After equilibration of the Sephadex PD MidiTRAP G10 (Cytiva MiniTrap G-10, #Cat: 28,918,011) with 20 mL of saline, 0.5-1 mL of the respective crude radiolabeling mixture was added to the cartridge. The liquid was allowed to sink completely into the bed. The required volume of eluent (0.9% NaCl) for the subsequent column wash was determined by subtracting the already added sample volume from 1.7 mL of total volume required. After collection of the wash eluate, up to 2 mL of saline were used to elute the respective radiolabeled peptide. For elution volumes ≥ 1.5 mL, the final pH of the eluate was adjusted to pH 6–7 by using phosphate buffer (0.5 M, 300 µL, pH 8).

#### Measurement of activity and recovery rates

Recovery rates were determined by measuring the residual activity on the column, the activity in the initial 1.7 mL column wash and the product eluate. An α-β-γ Raditec activimeter with an IBC-LITE software was used for activity quantification. All values were decay-corrected.

### Electronic supplementary material

Below is the link to the electronic supplementary material.

Supplementary Material 1

## Data Availability

All data generated and analysed during this study are included in this published article and its supplementary information.

## Abbreviations

SEC: Size exclusion chromatography
SPE: Solid phase extraction
RCP: Radiochemical purity
RCC: Radiochemical conversion
RP: Reverse phase
PSMA: Prostate specific membrane antigen
SCX: Solid cationic exchange
ITLC: Instant thin layer chromatography
Rf: Retention factor
HPLC: High performance liquid chromatography
Rt: Retention time
ACN: Acetonitrile
