# Supplementary material for: Validation of a size exclusion method for concomitant purification and formulation of peptide radiopharmaceuticals
Source: EJNMMI Radiopharm Chem. 2024 Mar 21;9:23. doi: 10.1186/s41181-024-00254-2 (PMC10957824; doi:10.1186/s41181-024-00254-2)
Supplement: Supplementary file 1 — Supplementary Material 1 [file 41181_2024_254_MOESM1_ESM.docx]

**Supporting Information**

**Validation of a size exclusion method for concomitant purification and formulation of peptide radiopharmaceuticals**

Sebastian Martin^1,2,3^, Lennard Wendlinger^1,2,3^, Alexandra Litvinenko^1,2,3^, Radmila Faizova^1,2,3^, Margret Schottelius^1,2,3,*^

1. Translational Radiopharmaceutical Sciences, Department of Nuclear Medicine and Department of Oncology, Centre Hospitalier Universitaire Vaudois (CHUV) and University of Lausanne (UNIL), 1011 Lausanne, Switzerland
2. AGORA, Pôle de recherche sur le cancer, 1011 Lausanne, Switzerland
3. SCCL Swiss Cancer Center Leman, 1011 Lausanne, Switzerland

***Corresponding Author**

Prof. Dr. Margret Schottelius

Translational Radiopharmaceutical Sciences

Department of Nuclear Medicine, CHUV

Department of Oncology, UNIL

Rue du Bugnon 25A, Agora

CH-1011 Lausanne

Switzerland

Phone +41.21.545.1120

Mobile +41.79.556.0143

Email: margret.schottelius@chuv.ch

**Contents**

Figure S1 Structure of PSMA-HSG. 3

Figure S2 Structure of PSMA-617. 3

Figure S3 Structure of hPep-1-DOTA. 4

Figure S4 Structure of mPep-1-DOTA. 4

Figure S5 Structure of DOTA-RAP-103. 5

Figure S6 Structure of NO-Y-103. 5

Figure S7 Structure of TRAP-103. 6

Figure S 8 Analytical chromatogram of radiotracer [^68^Ga]Ga-TRAP-103. 7

Figure S 9 Analytical chromatogram radiotracer [^68^Ga]Ga-DOTA-RAP-103. 7

Figure S 10 Analytical chromatogram of radiotracer [^64^Cu]Cu-NO-Y-103. 7

Figure S 11 Analytical chromatogram of radiotracer [99mTc]Tc-PSMA-HSG. 7

Figure S12 Scheme of a semi-automated implementation of a G10 column on a Scintomics GRP V3 synthesizer module. 8

[Table S1 Investigated elution buffers of ^68^Ga-labeled peptides using a G10 column. 8](#_Toc156146920)

Figure S1 Structure of PSMA-HSG.

Figure S2 Structure of PSMA-617.

Figure S3 Structure of hPep-1-DOTA.

Figure S4 Structure of mPep-1-DOTA.

Figure S5 Structure of DOTA-RAP-103.

Figure S6 Structure of NO-Y-103.

Figure S7 Structure of TRAP-103.

Figure S 8 Analytical chromatogram of radiotracer [^68^Ga]Ga-TRAP-103. Chromolith column, gradient of 10-70% B in 15 min., R_t_ 3.4min, RCP >99%.

Figure S 9 Analytical chromatogram radiotracer [^68^Ga]Ga-DOTA-RAP-103. MultiChrom column, gradient of 5-50% B in 10 min., R_t_ 8.1 min, RCP >99%.

Figure S 10 Analytical chromatogram of radiotracer [^64^Cu]Cu-NO-Y-103. Chromolith column, gradient of 10-70% B in 15 min., R_t_ 3.8 min, RCP >99%.

Figure S 11 Analytical chromatogram of radiotracer [99mTc]Tc-PSMA-HSG. Typical double peak observed by the two conformations formed with the mas_3_ chelator. MultoKrom column, Gradient 10-90% B in 15 min, R_t_ 9.7 and 10.1 min, RCP >99%.


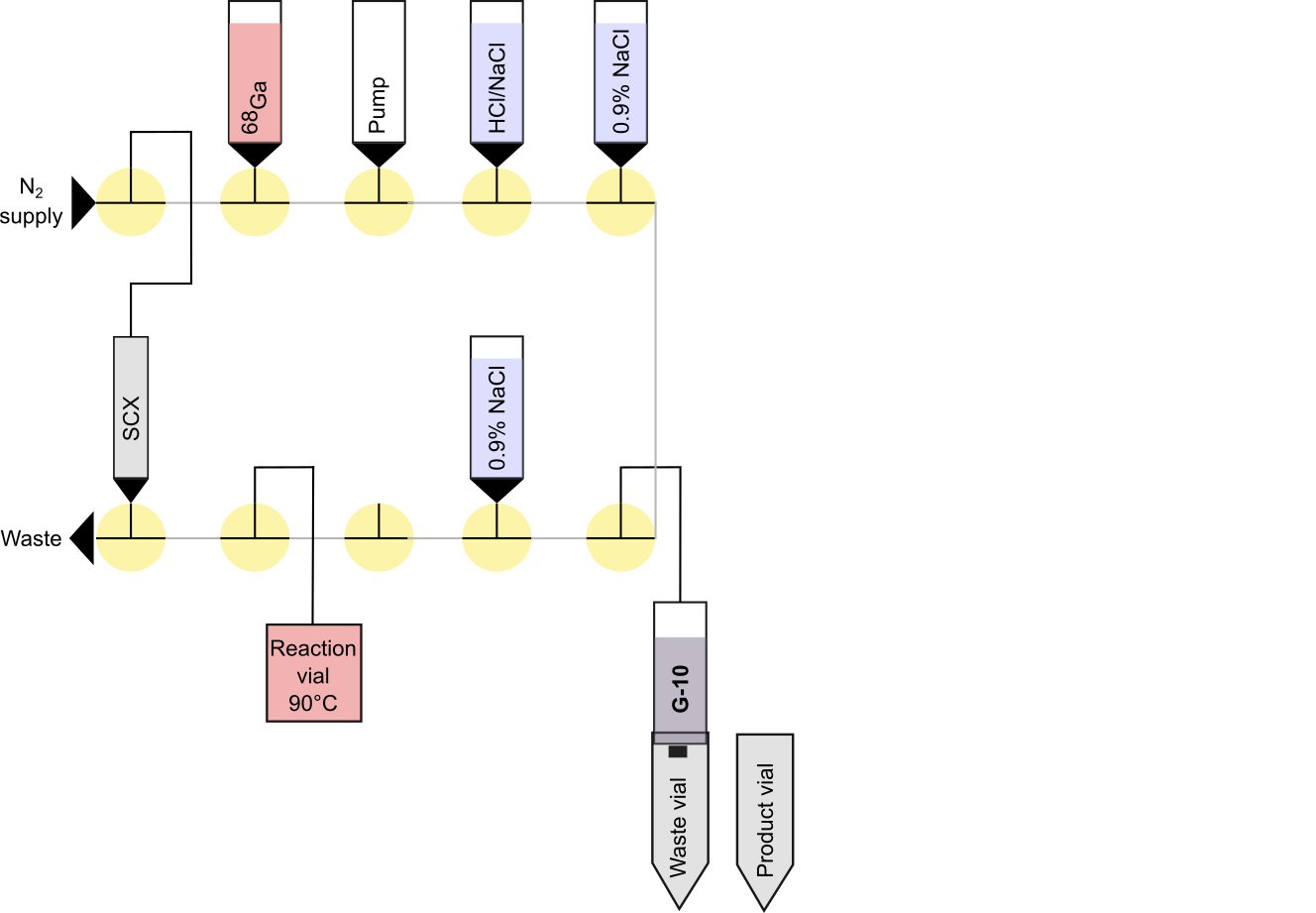


Figure S 12 Scheme of a semi-automated implementation of a G10 column on a Scintomics GRP V3 synthesizer. Step1: The ^68^Ga is eluted in 0.1M HCl and passed through the activated SCX cartridge. Step 2: The ^68^Ga is eluted with 0.7-0.75 mL HCl/NaCl into the reaction vial containing 5-10nmol the radiotracer precursor and 350 μL 1M NaOAc. Step 3: The reaction vial is heated for 5 min at 90°C. Step 4: The crude is added to the column. Step 5: The wash is performed with 0.7 mL of 0.9% NaCl. Step 6: After changing to the product vial the product is eluted using 1.2 mL of 0.9% NaCl. Overall synthesis time 35 minutes.

Table S1 Investigated elution buffers of ^68^Ga-labeled peptides using a G10 column for the radiotracer work-up.

|  |  | [^68^Ga]Ga-PSMA617 | | [^68^Ga]Ga-TRAP-103 | |
| --- | --- | --- | --- | --- | --- |
| Utilized buffers | Supplemented agent | elution vol. [mL] | Recovery [%] | elution vol. [mL] | Recovery [%] |
| 0.9% NaCl | - | 1.2 | 67.1±2.9 | 1.2 | 90.4±4.7 |
| 0.9% NaCl | - | 1.5 | 75.5±1.4 | 1.5 | 93.2±2.4 |
| 0.9% NaCl | - | 2 | 83.6±1.2 | - | - |
| 0.9%NaCl | 5%EtOH | 2 | 81.0 | 1.5 | 90.8 |
| 0.9%NaCl | 50mM Guanidine | 1.2 | 66.3 | 1.5 | n/a |
| 0.9%NaCl | 0.5% PS80 | 1.2 | 62.8 | 1.5 | 90.1 |
